# Le syndrome d'ogilvie post césarienne: une complication mystérieuse: à propos d'un cas

**DOI:** 10.11604/pamj.2014.19.368.4159

**Published:** 2014-12-10

**Authors:** Sarah Amourak, Mariam Tayae, Sofia Jayi, Fatimazahra Fdili Alaoui, Hakima Bouguern, Hikmat Chaara, Moulay Abdelilah Melhouf

**Affiliations:** 1 Université Sidi Mohammed Benabdellah, Service de Gynécologie-Obstétrique 2, CHU Hassan II de Fès, Maroc

**Keywords:** Syndrome d′ogilvie, césarienne, distension cæcale, perforation cæcale, prostigmine, Ogilvie syndrome, Caesarean section, cecal distension, cecal perforation, prostigmine

## Abstract

Le syndrome d'ogilvie ou appeler encore Pseudo-occlusion colique aiguë (acute colonic pseudo-obstruction), décrit par Sir William Ogilvie en 1948 et correspond à une dilatation aiguë du colon antérieurement sain, survenant en l'absence d'obstruction mécanique avec un diamètre cæcal > 9 cm, La symptomatologie correspond à celle d'une occlusion intestinale basse, d'installation rapide et l'imagerie fait d'ASP et de TDM abdominale permet de mettre en évidence la distension cæcale dont la mesure de son diamètre permet de prédire le risque de perforation. A travers notre cas et une revue de la littérature nous invitons les obstétriciens à ne pas méconnaitre ce syndrome car seul un diagnostic précoce permet de réduire le risque de perforation cæcale

## Introduction

Le syndrome d'ogilvie ou appeler encor Pseudo-occlusion colique aiguë (acute colonic pseudo-obstruction), décrit par Sir William Ogilvie en 1948 et correspond à une dilatation aiguë du colon antérieurement sain, survenant en l'absence d'obstruction mécanique avec un diamètre cæcal > 9 cm.

## Patient et observation

A travers notre cas et une revue de la littérature, nous essayerons de préciser les caractéristiques diagnostiques, thérapeutiques, pronostiques de cette affection dont La prise en charge repose sur une reconnaissance rapide du diagnostic. C'est une partiente de 29 ans, primigeste, sans antécédents pathologiques notables, césarisée pour suspicion de souffrance foetale aiguë en début du travail, qui s'est déroulé sous rachianesthésie.

**Figure 1:**
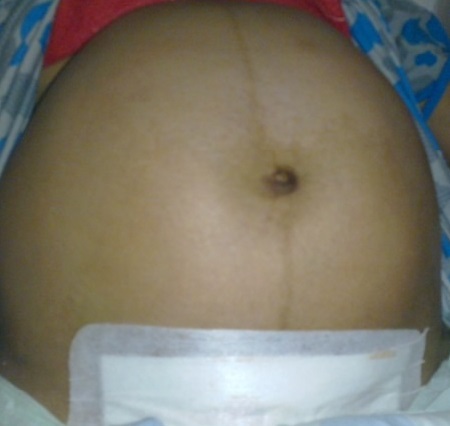
Énorme distension abdominale à J0 post césarienne

La patiente a présenté à J0 une distension abdominale diffuse s'aggravant progressivement avec des vomissements (Figure 1), l'examen clinique trouve une patiente fébrile à 39°C, tachycarde avec un abdomen très distendu, tympanique, une sensibilité épigastrique a été constaté et au toucher rectal l'ampoule rectale vide. Elle a bénéficié d'un ASP qui n'a pas montré de niveaux hydroaériques, l’échographie abdominale a objectivé une distension colique de 5cm au niveau du cæcum avec une fine lame d’épanchement intrapéritonéal. La TDM abdominale a constaté une importante distension cæcale à 9cm et de la dernière anse iléale.

Sur le plan biologique elle a présenté une hyperleucocytose à 17000 et une CRP à 338. La patiente a été transférée au service de réanimation, ou elle a bénéficié d'une sonde nasogastrique et d'une sonde rectale, une bonne réhydratation, elle a été mise sous atropine et prostigmine pour relancer le transit intestinal, avec une bonne évolution, la patiente a repris le transit à j + 2 et elle a été déclarée sortante à j + 9.

## Discussion

Chez la femme, la césarienne apparaît comme étant l'intervention chirurgicale la plus fréquemment associée au syndrome d'Ogilvie [1]. La physiopathologie de ce syndrome est incertaine, Les causes gynécologiques sont les plus fréquentes avec tout d'abord la césarienne, la grossesse, la chirurgie pelvienne suivies par les traumatismes [2]. Ces pathologies entraineraient une dysfonction des nerfs parasympatiques sacrés (S2, S3, S4), responsable d'une atonie du colon gauche et du rectum causant une occlusion fonctionnelle. Cette théorie est renforcée par la jonction entre colon normal et dilaté qui se situe le plus souvent au niveau de l'angle colique gauche. Le colon proximal dont les centres parasympatiques proviennent du nerf vague aurait un péristaltisme normal.

Le système sympathique aurait un rôle inhibiteur et le parasympatique un rôle excitateur. L'interaction entre ces deux systèmes régulerait l'activité colique [3]. Plus récemment, il a été proposé que l'inhibition colique était liée à une hypertonie sympathique. Cette hypertonie serait retrouvée chez les patients souffrant de diverses agressions pathologiques [4]. Les perturbations de la motricité colique ont aussi été rattachées à des désordres métaboliques provoquant un trouble de la conduction neuromusculaire (hypokaliémie, hyper-urémie) [5].

D'après les différentes publications, l'origine de ce syndrome semble être multifactorielle et survient dans des contextes autres que la période postopératoire [6]. La symptomatologie correspond à celle d'une occlusion intestinale basse, d'installation rapide (un à deux jours) ou plus lente en trois à sept jours, le météorisme et la distension abdominale étant les signes les plus fréquents. En l'absence de complications, l'abdomen reste souple, les bruits hydro-aériques sont normaux, voire augmentés et l’état général est le plus souvent conservé. L'arrêt des matières est uniquement présent dans la moitié des cas, les nausées et les vomissements sont inconstants [7, 8]. Les examens biologiques sont peu contributifs par contre L'imagerie permet de poser le diagnostic, l'examen à réaliser en première intention est un cliché d'abdomen sans préparation. Il montre une distension gazeuse colique globale s’étendant du cæcum à la jonction recto-sigmoïdienne La mesure du diamètre cæcal apparaît comme le meilleur indice prédictif du risque de perforation du cæcum. La tomodensitométrie abdominale avec injection de produit de contraste peut être réalisée et permet d’éliminer certains diagnostics différentiels tels que l'obstacle mécanique, le volvulus du sigmoïde ou du cæcum, le fécalome ou la péritonite. Le risque majeur est la perforation cæcale compliquée d'une péritonite stercorale même après plusieurs jours d’évolution favorable. Ce risque augmente avec un diamètre cæcal supérieur à 12 cm ou si la distension a duré plus de six jours. Le risque de perforation spontanée est d'environ 3% mais le taux de mortalité en cas de perforation est d'environ 40% [9].

Le traitement médical associe le jeûne, la pose d'une sonde naso-gastrique, la ré-équilibration des désordres électrolytiques, la suppression des facteurs favorisants et éventuellement des lavements distaux à l'aide d'une sonde rectale. Les traitements parasympathicomimétiques comme la prostigmine peuvent être utilisés en intraveineux, puis la néostigmine par voie intraveineuse est instaurée; elle a montré son efficacité même s'il existe un risque de récidive des symptômes. De plus l'administration de ce traitement du fait du risque de bradycardie, nécessite une présence. médicale lors de l'injection avec monitorage cardiotensionnel pendant 30 minutes et injection d'atropine disponible en cas d'effet indésirable. La coloscopie d'exsufflation peut être envisagé comme étant le premier geste « invasif » à réaliser. Le taux d’échec ou de récidive des symptômes est de plus de 30%; mais la mise en place d'un drain en cours de procédure améliore ce taux [6]. L’évolution peut se faire vers une stabilisation ou une diminution de la distension gazeuse spontanément ou après traitement, mais il existe un risque de récidive malgré plusieurs jours d’évolution favorable [10].

## Conclusion

Le syndrome d'ogilvie correspond à une dilatation aiguë du colon antérieurement sain, survenant en l'absence d'obstruction mécanique avec un diamètre cæcal >9 cm, La symptomatologie correspond à celle d'une occlusion intestinale basse, d'installation rapide et l'imagerie fait d'ASP et de TDM abdominale permet de mettre en évidence la distension cæcale dont la mesure de son diamètre permet de prédire le risque de perforation. A travers notre cas et une revue de la littérature nous invitons les obstétriciens à ne pas méconnaitre ce syndrome car seul un diagnostic précoce permet de réduire le risque de perforation cæcale.
